# The reference genome and abiotic stress responses of the model perennial grass *Brachypodium sylvaticum*

**DOI:** 10.1093/g3journal/jkad245

**Published:** 2023-10-26

**Authors:** Li Lei, Sean P Gordon, Lifeng Liu, Nir Sade, John T Lovell, Maria Del Mar Rubio Wilhelmi, Vasanth Singan, Avinash Sreedasyam, Rachel Hestrin, Jeremy Phillips, Bryan T Hernandez, Kerrie Barry, Shengqiang Shu, Jerry Jenkins, Jeremy Schmutz, David M Goodstein, Roger Thilmony, Eduardo Blumwald, John P Vogel

**Affiliations:** U.S. Department of Energy Joint Genome Institute, Lawrence Berkeley National Laboratory, Berkeley, CA 94720, USA; U.S. Department of Energy Joint Genome Institute, Lawrence Berkeley National Laboratory, Berkeley, CA 94720, USA; U.S. Department of Energy Joint Genome Institute, Lawrence Berkeley National Laboratory, Berkeley, CA 94720, USA; Department of Plant Sciences, University of California, Davis, CA 95616, USA; School of Plant Sciences and Food Security, Tel Aviv University, Tel Aviv 69978, Israel; U.S. Department of Energy Joint Genome Institute, Lawrence Berkeley National Laboratory, Berkeley, CA 94720, USA; Genome Sequencing Center, HudsonAlpha Institute for Biotechnology, Huntsville, AL 35806, USA; Department of Plant Sciences, University of California, Davis, CA 95616, USA; U.S. Department of Energy Joint Genome Institute, Lawrence Berkeley National Laboratory, Berkeley, CA 94720, USA; Genome Sequencing Center, HudsonAlpha Institute for Biotechnology, Huntsville, AL 35806, USA; U.S. Department of Energy Joint Genome Institute, Lawrence Berkeley National Laboratory, Berkeley, CA 94720, USA; U.S. Department of Energy Joint Genome Institute, Lawrence Berkeley National Laboratory, Berkeley, CA 94720, USA; Crop Improvement and Genetics Research Unit, USDA-ARS Western Regional Research Center, Albany, CA 94710, USA; U.S. Department of Energy Joint Genome Institute, Lawrence Berkeley National Laboratory, Berkeley, CA 94720, USA; U.S. Department of Energy Joint Genome Institute, Lawrence Berkeley National Laboratory, Berkeley, CA 94720, USA; Genome Sequencing Center, HudsonAlpha Institute for Biotechnology, Huntsville, AL 35806, USA; U.S. Department of Energy Joint Genome Institute, Lawrence Berkeley National Laboratory, Berkeley, CA 94720, USA; Genome Sequencing Center, HudsonAlpha Institute for Biotechnology, Huntsville, AL 35806, USA; U.S. Department of Energy Joint Genome Institute, Lawrence Berkeley National Laboratory, Berkeley, CA 94720, USA; Crop Improvement and Genetics Research Unit, USDA-ARS Western Regional Research Center, Albany, CA 94710, USA; Department of Plant Sciences, University of California, Davis, CA 95616, USA; U.S. Department of Energy Joint Genome Institute, Lawrence Berkeley National Laboratory, Berkeley, CA 94720, USA

**Keywords:** genome, perennial grass, transcriptome, abiotic stress, transposable element, Plant Genetics and Genomics

## Abstract

Perennial grasses are important forage crops and emerging biomass crops and have the potential to be more sustainable grain crops. However, most perennial grass crops are difficult experimental subjects due to their large size, difficult genetics, and/or their recalcitrance to transformation. Thus, a tractable model perennial grass could be used to rapidly make discoveries that can be translated to perennial grass crops. *Brachypodium sylvaticum* has the potential to serve as such a model because of its small size, rapid generation time, simple genetics, and transformability. Here, we provide a high-quality genome assembly and annotation for *B. sylvaticum*, an essential resource for a modern model system. In addition, we conducted transcriptomic studies under 4 abiotic stresses (water, heat, salt, and freezing). Our results indicate that crowns are more responsive to freezing than leaves which may help them overwinter. We observed extensive transcriptional responses with varying temporal dynamics to all abiotic stresses, including classic heat-responsive genes. These results can be used to form testable hypotheses about how perennial grasses respond to these stresses. Taken together, these results will allow *B. sylvaticum* to serve as a truly tractable perennial model system.

## Introduction

Perennial grasses are grown throughout the world for forage and turf. In addition, they have the potential to become a major source of renewable energy. Compared to annuals, perennial grasses have significant agronomic advantages. Since they are not planted every year, perennials require less energy input which leads to a more favorable net carbon balance. Perennials begin growth in the spring as soon as conditions are favorable and, depending on the species, can grow until frost. This allows them to intercept more sunlight over the growing season which can lead to greater biomass production and decreased competition with weeds. On average, perennial crops have higher water use efficiency and need less fertilizer than annual crops (Cosentino et al. 2007; Monti and Zatta 2009; Cosentino et al. 2014). Cultivation of perennial crops can also increase soil carbon content which may reduce atmospheric carbon (Chimento and Amaducci 2015). Perennial grasses (like switchgrass) are essential components of the North American tallgrass prairie which was one of the largest temperate biomes on Earth (Searle and Malins 2014). Thus, large-scale growth of native perennial grasses for biofuel or other uses may benefit wildlife and local ecosystems.

Perenniality requires the optimization of a suite of developmental, physiological, and environmental response traits (Lundgren and Des Marais 2020). At a minimum, perennials in temperate regions require the ability to continue vegetative growth after flowering and the ability to survive over the winter. Additional traits that enhance the fitness of perennials include optimizing sink–source relationships between reproduction, vegetative growth, and overwintering organs; controlled senescence and nutrient remobilization; resource allocation to defenses to extend lifespan without decreasing long-term fitness; and sensing and responding to environmental cues to optimize the timing of growth, reproduction, and dormancy. These traits all exist on a continuum, and where a plant falls on that continuum will determine if it is an annual, a weak perennial, or a long-lived perennial. Additionally, these traits also affect the productivity of a plant in an agricultural ecosystem and have been selected for during the development of our crops, both annual and perennial. Thus, a mechanistic understanding of the molecular control of these traits would be useful for the rational development of perennial crops for various applications, e.g. grain production, biomass production, and carbon sequestration. Unfortunately, knowledge of the molecular mechanisms underlying these traits in perennials is rudimentary.

Although perennial grasses are important forage, turf, and emerging biomass crops, they are challenging to study for several reasons. Their large size and slow generation time make them difficult to study under controlled conditions. In addition, most forage grasses and the grasses emerging as biomass crops are genetically challenging because of their large polyploid genomes, outcrossing nature, and/or vegetative reproduction. Finally, efficient transformation systems do not exist for most perennial grass crops which severely limit functional genomics. Therefore, a model system is required to understand the molecular mechanisms underlying perenniality, productivity, and abiotic stress tolerance in perennial grasses.

Abiotic factors such as temperature, drought, and salt can limit both grain and biomass yield. Indeed, abiotic stress has been estimated to reduce the worldwide yield of major crops by 9.4 billion tons in 2019 according to the FAO Statistical Yearbook 2021 (FAO 2021). While abiotic stress is important for all crops, it is particularly important for biomass crops because they should be grown on marginal land to avoid competition with food crops. Fortunately, since the perennial grasses under consideration for use as biomass crops (e.g. switchgrass) are not fully domesticated, there is enormous opportunity to improve traits like stress tolerance and nutrient use efficiency. Knowledge of transcriptional responses to abiotic stresses is an important step toward understanding and improving stress tolerance. For example, analysis of the transcriptional responses of *Arabidopsis* seedlings to *Botrytis cinerea* infection and drought, along with mutant analysis, identified *RD20* as a stress-responsive gene that confers some resistance to both drought and *B. cinerea* (Sham et al. 2015). Another example is the use of transcriptomic data from maize seedlings and heterologous expression in *Arabidopsis* to demonstrate that *ZmWRKY106* is involved in multiple abiotic stress response pathways and can increase tolerance to drought and heat stress (Wang et al. 2018a). These genes are candidates for biotechnological manipulation or breeding targets to increase stress tolerance. Therefore, characterizing the transcriptional responses in a model system could help develop testable hypotheses and develop approaches to improve stress tolerance.

*Brachypodium sylvaticum* is a long-lived perennial grass (Haeggstrom and Skyten 1996) that possesses many of the traits (e.g. diploidy, self-fertility, small genome size, rapid generation time, and small stature) that have made its congener *Brachypodium distachyon* a valuable model for annual grasses (Draper et al. 2001; Hasterok et al. 2022). Since *B. distachyon* and *B. sylvaticum* are closely related, the knowledge and protocols created for *B. distachyon* can be leveraged to accelerate the development of the resources required to use *B. sylvaticum* as a model. For example, by using the *B. distachyon* transformation protocol (Bragg et al. 2015) as a starting point, a highly efficient *B. sylvaticum* transformation protocol with an average efficiency of 67% was developed (Steinwand et al. 2013). In addition, we genetically characterized 15 inbred lines to provide a nucleus for natural diversity studies (Steinwand et al. 2013). A large sequencing project that will create a *B. sylvaticum* pan-genome is underway at the DOE Joint Genome Institute (https://jgi.doe.gov/brachypodium-model-grass-genus-bioenergy/). Previous research based on phenotypic monitoring and expression analysis demonstrated that *B. sylvaticum* exhibits freezing tolerance that is enhanced by cold acclimation (Gordon et al. 2015; Toubiana et al. 2020). However, these are just the first steps toward developing a global understanding of stress responses in this model grass.

While *B. sylvaticum* possesses the biological traits and some of the experimental resources necessary for a modern model plant, it is lacking a reference genome sequence. To fill this void, we created a high-quality reference genome and annotation. We placed this genome into evolutionary context through syntenic comparisons to other grasses and phylogenetic analysis with broader eukaryotes. In addition, we conducted transcriptomic analysis to better understand responses to water, salt, heat, and cold stresses. Our results provide vital resources for this model system and begin to build a framework of how *B. sylvaticum* responds to abiotic stress.

## Materials and methods

### Sequencing and assembly

To reduce heterozygosity, *B. sylvaticum* line Ain1 was inbred through 7 generations of single seed descent. High molecular weight DNA from young leaves (4- or 5-leaf stage) was isolated using a published protocol (Peterson et al. 2000). Large insert libraries were prepared using the 20 kb insert PacBio protocol and sequenced to greater than 60× depth on the PacBio RS II system with P6-C4 chemistry. Raw PacBio reads (BioProjectID PRJNA786589) were processed and assembled with the Falcon genome assembler (release as of 2015 September 12; Chin et al. 2013, 2016). The resulting contigs were subsequently processed with 2 rounds of error correction using Quiver (Chin et al. 2013) followed by additional error correction using finisherSC (Lam et al. 2015). A base accuracy of less than 1 error per 56 kb of genomic sequence was estimated by aligning Illumina data (BioProjectID PRJNA786589) generated from Ain1 to the Ain1 PacBio assembly using BWA mem (Li and Durbin 2009) and samtools (Li 2011).

### F_2_ mapping population and genetic map construction

An F_2_ mapping population consisting of 288 individuals was generated via a cross between the inbred lines Ain1 and Sin1. Ain1 and Sin1 were sequenced to greater than 50× mean depth with Illumina paired-end sequences on an Illumina HiSeq2500 instrument. Illumina sequences (BioProjectID PRJNA786589) from parental lines were compared with each other using 51-mers to identify single nucleotide variants that distinguish the 2 parental lines to use as markers. The markers at this step were just the 51-mer sequence plus the associated line tag (Ain1 or Sin1) with no coordinates. These markers were then aligned to the initial genome assembly with BWA mem and assigned coordinates based on the assembled contigs. The markers were subsequently used to genotype the F_2_ mapping population as described below.

DNA was isolated from individual plants from the F_2_ mapping population, and Illumina sequencing libraries were prepared using a reduced representation protocol consisting of digesting DNA with ApeKI restriction enzyme followed by ligating ApeKI compatible oligos to the cut fragments and PCR amplification of the ligated product to add barcoded Illumina sequencing adapters. PCR products were purified using magnetic beads. The concentration of each amplified library was estimated using a Qubit HS assay. Barcoded libraries were pooled and sequenced with single-end 100-bp reads on an Illumina HiSeq 2000 sequencer at the University of Wisconsin Biotechnology Center Sequencing Core. Resulting multiplexed sequencing reads (BioProjectID PRJNA786589) were demultiplexed using GBSX (Herten et al. 2015) using the known barcode adapter sequences. Demultiplexed Illumina reads for each of the 288 F_2_ individuals were then compared to the 51-bp genotyping markers above to determine the parental genotypes at each locus. Raw genotyping calls were improved by majority rule binning within 14-kb intervals to determine consensus genotypes within these windows tiling the genome using custom python scripts.

MSTMap (Wu et al. 2008) was used with a Kosambi distance function and count objective function to produce linkage groups and genetic distances from the consensus genotypes. Assembly errors were identified by looking for simultaneous changes in haplotype across most F_2_ lines in each contig. Three contigs were broken in this process. The contigs of the polished PacBio assembly were then oriented, ordered, and joined together into 9 chromosome-level pseudomolecules using the genetic linkage map produced by MST map.

### Annotation

Transcript assemblies were constructed from a diverse set of 73 paired-end Illumina RNA-seq libraries (Supplementary Table 1) using an internal JGI pipeline (PERTRAN) that includes the following steps: RNA-seq reads were first assembled using a reference-based transcript assembly workflow, and then those assembled transcript fragments were processed by PASA (Haas et al. 2003) to create the final transcript assembly set (129,529 transcript assemblies). Genic loci were determined by transcript assembly alignments and/or EXONERATE alignments of proteins from *B. distachyon*, *Brachypodium stacei*, *Arabidopsis thaliana*, *Glycine max*, *Sorghum bicolor*, *Oryza sativa*, *Setaria italica*, *Setaria viridis*, *Vitis vinifera*, and Swiss-Prot eukaryotic proteins (downloaded November 2016) to soft-repeatmasked *B. sylvaticum* Ain1 genome. The genome was masked using RepeatMasker (Smit et al. 1996) with the MIPS *B. distachyon* repeat library as well as a de novo repeat predictions from the *B. sylvaticum* genome using RepeatModeler (Flynn et al. 2020) with up to 2,000 bp extension on both ends without extending into another locus on the same strand. Gene models were predicted by homology-based predictors, FGENESH+ (Salamov and Solovyev 2000), FGENESH_EST (similar to FGENESH+ but with EST as splice site and intron input instead of protein/translated ORF), and GenomeScan (Yeh et al. 2001).

The best gene predictions for each locus were selected using multiple positive factors, including EST and protein support, and one negative factor: overlap with repeats. The selected gene predictions were improved by PASA (Haas et al. 2003). Improvement included adding UTRs, splicing correction, and adding alternative transcripts. PASA-improved gene model proteins were subject to protein homology analysis using the proteomes mentioned above to obtain C score and protein coverage. C score is a ratio of protein BLASTP score to mutual best hit BLASTP score, and protein coverage is the highest percentage of protein aligned to the best homologs. PASA-improved transcripts were selected based on C score, protein coverage, EST coverage, and its CDS overlapping with repeats. The transcripts were selected if its C score is ≥ 0.5 and protein coverage ≥ 0.5, or if it has EST coverage, but its CDS overlaps < 20% with repeats. For gene models whose CDS overlaps with repeats for more than 20%, its C score must be at least 0.9 and homology coverage at least 70% to be selected. The selected gene models were subject to Pfam analysis, and gene models whose protein were more than 30% in Pfam TE domains were removed. Incomplete gene models, low homology supported without fully transcriptome supported gene models and short single exon (<300 bp CDS) without protein domain, nor good expression gene models were manually filtered out.

### Synteny analysis

The genomes of 6 species, including 2 perennial species: *B. sylvaticum* (*B. sylvaticum* v1.1) and *Panicum hallii* (*P. hallii* HAL v2.2), and 4 annual species: *B. distachyon* (*B. distachyon* v3.2), *B. stacei* (*B. stacei* v1.1), *S. bicolor* (*S. bicolor* v3.1.1), and *O. sativa* (*O. sativa* Kitaake v3.1), were downloaded from Phytozome 13 (https://phytozome-next.jgi.doe.gov/; Goodstein et al. 2012). We used a comparative genomic approach to determine syntenic relationships among the 5 genomes to accomplish the following goals: (1) identify orthologous pairs of genes, (2) define the ortholog gene families, and (3) understand the scale of synteny among the 5 grass species. Given these goals and the highly repetitive and poorly conserved intergenic regions in plant genomes, we used a gene-level approach to do whole-genome alignments. We ignored regions that were not in proximity to annotated gene models. The GENESPACE pipeline from Lovell et al. (2018) was applied to perform syntenic analysis. In short, GENESPACE conducts standard inference of orthology using the OrthoFinder program (Emms and Kelly 2019) but limits the search within known colinear (syntenic) blocks, generated by the multiple collinearity inference program MCScanX42 (Wang et al. 2012b). This allows for the inference of orthology in duplicated chromosomal regions, as these appear as multiple distinct blocks in the alignments. In addition to pairwise peptide–peptide searches for orthologous gene groups, GENESPACE also conducts alignments against unannotated genome sequences via BLAT (Kent 2002) and EXONERATE (Slater and Birney 2005) to discover the sequence identity of pseudogenized or otherwise unannotated loci. The pipeline outputs alignments and some general sequence divergence statistics for all orthogroup sequences among all genomes considered.

### Transcriptome sequencing and expression analysis

Seeds from fifth generation inbred *B. sylvaticum* Ain1 plants were used for this study. Some experiments were conducted for both annotation and expression analysis, and some were only used for annotation. Samples were sequenced at 3 different facilities as indicated in Supplementary Table 1. To avoid confounding factors, only data produced at a single center at the same time was compared in our transcriptional analyses. For the abiotic stress time course experiment (Supplementary Fig. 2b), seeds were planted and stratified at 4°C for 1 week. Following stratification, pots were transferred to a long-day growth chamber (16 h/8 h day/light, 24°C/18°C, and 150 μmol/m^2^/s light intensity). After 20 days, plants were exposed to heat, drought, or salt and sampled at 1, 2, 5, 10, and 24 h after exposure to the abiotic stress. For water stress, plants were removed from soil and allowed to desiccate under ambient growth chamber conditions. For salt stress, soil was saturated with 500 mM NaCl. For heat exposure, plants were moved to a 42°C chamber with the same light cycle and intensity. After harvest, shoot RNA was extracted with the RNeasy Mini Kit coupled with an on-column DNase digestion (Qiagen).

For cold and freezing treatment (Supplementary Fig. 2a), plants were grown in a growth chamber for 21 days as described in Toubiana et al. (2020). Briefly, plants were grown at 16 h/8 h day/light, 26°C/18°C, and 300 μmol/m^2^/s light intensity and then moved to a cold room (10 h/14 h day/light, 4°C/4°C, and 300 μmol/m^2^/s light intensity) for 14 days to allow them to acclimate. The end of this period is defined as freeze 0 h and labeled as “cold” in the results. Then the cold acclimated plants were transferred to a growth chamber for freezing treatment, −6°C for 8 h without light, and labeled as “freeze,” followed by 4°C for 24 h without light and labeled as “recovery.” Shoots and crowns were harvested separately at 3 time points: “cold,” “freeze,” and “recovery” (Supplementary Fig. 2a). For this study, crowns are the part of the plant connecting the shoots and roots and were harvested by cutting off as much of the shoots and roots as possible. RNA was extracted with PureLink RNA mini kit (Invitrogen) and treated with Turbo DNA-free kit (Ambion) to remove contaminated DNA.

For gene annotation purposes, leaves, crowns, shoots, and roots were harvested at day 21 (∼4-leaf stage) and mature leaves, crowns, shoots, roots, nodes, second internodes, and flag leaves were harvested around day 60 when the first spike appeared. The second internodes were cut into 3 parts (bottom, middle, and top). Floral tissues (pistil, stamen, and lemma/palea) were harvested from 70–90-day-old plants with inflorescences prior to or just beginning anthesis. Spikelet tissues were dissected with forceps and immediately frozen in liquid nitrogen. Plants were also grown with different forms of nitrogen (ammonium, nitrate, or urea). The tissues were ground to a fine powder, and RNA was extracted with the RNeasy Mini Kit coupled with an on-column DNase digestion (Qiagen).

The RNA quantity and quality were checked with Qubit RNA BR kit (Invitrogen), NanoDrop 1000 (Thermo Fisher Scientific), and RNA 6000 Nano kit and Bioanalyzer (Agilent). RNA sequencing for gene annotation was done at the UC Berkeley Sequencing Core and the UC Davis Sequencing Core with paired-end Illumina while all the stress experiments were sequenced at JGI. Isolated RNA was subject to library preparation with TruSeq Stranded mRNA Library Preparation Kit (Illumina) and then subjected to 150 bp paired-end sequencing with the Illumina HiSeq 2000 platform.

All RNA-seq raw reads were filtered and trimmed using the JGI QC pipeline (Singan, unpublished) resulting in the filtered fastq files. Using BBDuk (https://jgi.doe.gov/data-and-tools/software-tools/bbtools/), raw reads were evaluated for sequence artifacts by *k*-mer matching (*k*-mer = 25), allowing 1 mismatch, and artifacts were trimmed from the 3′ end of the reads. RNA spike-in reads, PhiX reads, and reads containing any Ns were removed. Quality trimming was performed using the phred trimming method set at Q6. Finally, following trimming, reads under the length threshold were removed (minimum length 25 bases or one-third of the original read length—whichever is longer). Filtered reads from each library were aligned to the reference genome using HISAT version 0.1.4-beta (Kim et al. 2015). The raw gene counts were generated using FeatureCounts (Liao et al. 2014) with gff3 annotations (described by above methods). Only primary hits assigned to the reverse strand were included in the raw gene counts (-s 2 -p --primary options). Raw gene counts were used to evaluate the level of correlation between biological replicates using Pearson’s correlation and determine which replicates would be used in the differential gene expression analysis. Raw data were deposited to SRA, and the accession numbers are included in Supplementary Table 1.

The DESeq2 package (Love et al. 2014) was used to perform pairwise comparisons between samples to identify differentially expressed genes (DEGs) using 2 criteria: the fold change (log_2_) had to be ≥ 1 or ≤ −1 and the adjusted *P*-value from DESeq analyses had to be < 0.05. The code used to identify DEGs and plot Venn diagrams is available at https://github.com/lilei1/B_sylvaticum.

From the genes with significant differential expression in ≥ 1 comparisons, we selected those with high variance and expression (variance >1.5 and mean expression >4) for further analysis. A priori identification of the number of clusters was performed using the sum of squared error (SSE) and hierarchical clustering. Then we performed *k*-mean clustering along the time course and calculated a membership score for each gene. We plotted the results for each gene with the core overlayed.

For the enrichment analysis, we downloaded all the *B. distachyon* WRKY and APETALA2 (AP2) members from PlantTFDB (http://planttfdb.gao-lab.org/download.php; Jin et al. 2014, 2015, 2017; Tian et al. 2019) and identified their *B. sylvaticum* orthologs. The WRKY gene family contains 121 members in *B.* sylvaticum (Supplementary Table 6), which is larger than both the *Arabidopsis* and rice WRKY gene families that contain 74 and 109 members, respectively (Phukan et al. 2016).

### Repetitive DNA analysis

De novo searches for long terminal repeat (LTR) retrotransposons used the output from the LTR_FINDER_parallel (Ou and Jiang 2019) and LTR to feed the LTR_Retriever (Ou and Jiang 2018). De novo detection of CACTA-DNA transposons and MITEs used custom programs (https://github.com/lilei1/B_sylvaticum). Known repeats were identified by RepeatMasker (Open-3-1-8; Smit et al. 1996) with the appropriate TE library.

### Gene Ontology analysis

Gene Ontology (GO) enrichments were identified using the AgriGO analysis toolkit in combination with the *B. distachyon* database (http://systemsbiology.cau.edu.cn/agriGOv2/; Tian et al. 2017). First, custom scripts were used to assign GO terms to *B. sylvaticum* genes based on their orthology to *B. distachyon* genes with assigned GO terms. The *B. sylvaticum* gene lists were then input into AgriGO to identify enrichments using Fisher's exact test and Benjamini–Hochberg False Discovery Rate (FDR) with a significance cutoff of FDR < 0.05.

### Phylostrata analysis

Gene ages were inferred using previously described methods (https://github.com/AlexGa/Phylostratigraphy; Drost et al. 2015). The NR protein database was downloaded from NCBI (2021 March 28), and all protein sequences were grouped according to 13 taxonomic levels [phylostrata (PS) 1: cellular organisms; PS2: Eukaryota; PS3: Viridiplantae; PS4: Streptophyta, Streptophytina; PS5: Embryophyta; PS6: Tracheophyta, Euphyllophyta; PS7: Spermatophyta; PS8: Magnoliophyta, Mesangiospermae; PS9: Liliopsida, Petrosaviidae, Commelinids, Poales; PS10: Poaceae; PS11: BOP clade; PS12: Pooideae, Brachypodieae, *Brachypodium*; and PS13: *B. sylvaticum*] based on NCBI taxonomy. *Brachypodium sylvaticum* genes were then mapped on the phylogeny using BLASTp (BLAST version 2.2.24) searches. We used 1 × 10^−5^ as the cutoff as has been used in previous publications (Quint et al. 2012; Drost et al. 2015; Wang et al. 2018b). *Brachypodium sylvaticum* genes were assigned to the lowest PS level at which they had a BLASTp hit below 10^−5^. More details can be found in https://github.com/lilei1/B_sylvaticum.

## Results and discussion

### Genome assembly and annotation

To increase the utility of *B. sylvaticum* as a model perennial grass, we produced a high-quality reference genome. The genome of inbred line Ain1 was sequenced using large insert PacBio libraries to ∼60× depth. The reads were assembled with the Falcon genome assembler (Chin et al. 2013, 2016) and error corrected with Quiver for 2 rounds (Chin et al. 2013). The resulting assembly contained 1,117 contigs with a contig N50 of 877 kb (Table 1). A genetic map based on a cross between inbred lines Ain1 and Sin1 was used to validate, order, and orient the contigs into pseudomolecules. Two hundred and eighty-eight F_2_ plants were genotyped using a genotyping-by-sequencing approach, and the resulting data were used to order the markers into 9 linkage groups corresponding to the 9 *B. sylvaticum* chromosomes. The final assembly consists of 9 pseudomolecules that contain 99.98% of the assembled sequence (Table 1). The assembly statistics compare favorably to other reference grass genomes (Table 1). The assembled genome (v1.1) and annotation is available through the Phytozome genome database (https://phytozome-next.jgi.doe.gov/info/Bsylvaticum_v1_1).

**Table 1.** Comparison of assembly and annotation features of the *B. sylvaticum* genome to other grasses.

Genome features	*B. sylvaticum* Ain1 (v1.1)	*B. distachyon* Bd21-3 (v3.2)	*B. stacei* ABR114 (v1.1)	*S. bicolor* BTx623 (v3.1.1)	*O. sativa* Kitaake (v3.1)	*P. hallii* HAL (v2.2)
Assembled genome size (Mb)	358.28	271.16	234.14	708.86	381.57	487.47
Scaffold total	15	10	112	175	33	43
Contig total	1,117	34	2,425	513	476	144
Scaffold sequence total (Mb)	509.2	271.2	234.1	677.7	381.6	487.5
Contig sequence total (Mb)	503.1	270.7	231.5	674.3	377.6	486.5
Scaffold N/L50 (Mb)	5/30.6	3/59.1	5/23.1	5/71.2	6/30.3	4/58.2
Contig N/L50 (Mb)	0.844/0.877	5/22	0.294/0.225	6/50.7	1.4/75	8.3/15
Number of scaffolds >50 kb	15	5	10	5	19	29
BUSCO score (%)	97.7	98.6	98.6	98.3	99.1	98.9
GC content (%)	46.37	46.32	44.71	41.81	43.15	46.84
Transposable elements (%)	39.72	23.06	16.15	61.16	45.29	53.21
Predicted protein-coding genes	36,927	34,310	29,898	35,490	35,596	33,805
Protein-coding transcripts	50,263	42,868	36,357	47,121	48,494	42,523
No. of transcripts per gene	1.36	1.25	1.22	1.33	1.36	1.26
Genes/Mb	103	127	128	50	93	69
Total coding sequence (Mb)	41.79	31	35.93	35.46	39.86	37.52
Average length of CDs per gene (bp)	1,131.57	903.4	1,201.61	999.15	1,119.89	1,109.78
Total intron sequence (Mb)	56.83	42.87	49.86	55.98	55.76	44.56
Average length of introns per gene (bp)	1,539.07	1,249.51	1,667.54	1,577.37	1,566.6	1,318.14

**Fig. 1.**
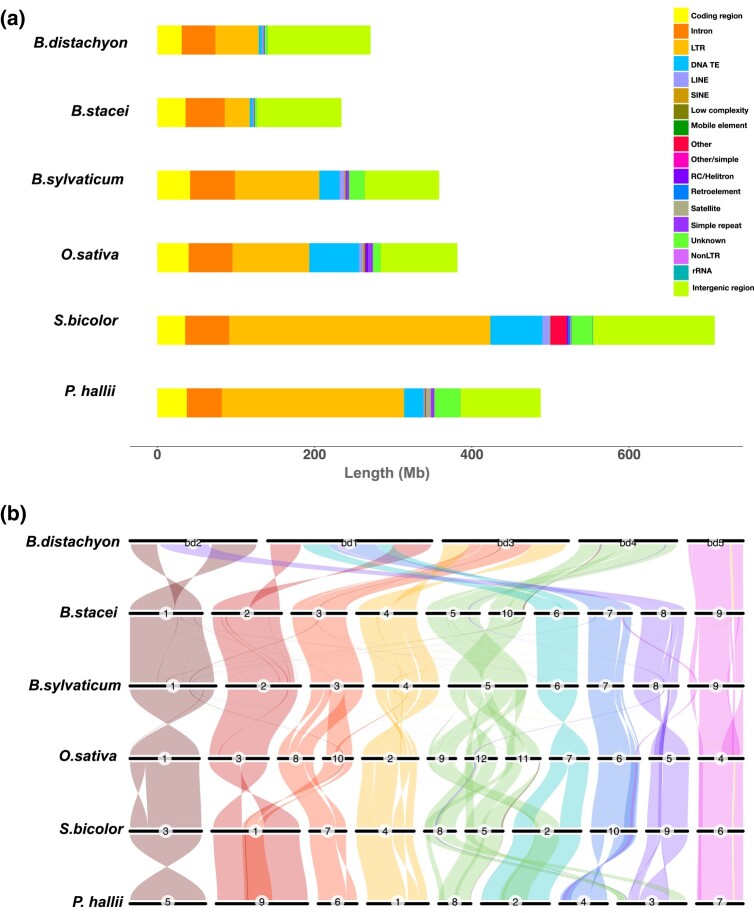
Genome analysis and synteny. a) The total length of genomic features in 6 grass genomes. b) Syntenic relationships between *B. sylvaticum* and 5 other diploid grasses: *B. distachyon*, *B. stacei*, *O. sativa*, *P. hallii*, and *S. bicolor*.

In eukaryotes, especially in higher plants, 2 major factors determine the accuracy of annotations: the quality of genome assembly and the availability of transcriptomic data from diverse tissues/treatments/timepoints to provide the evidence necessary to create and support accurate gene models (Salzberg 2019). Using the high-quality genome assembly described above, RNA-seq data from 32 different tissues/treatments/stages (see *Materials and methods*; Supplementary Table 1), and known plant proteins from other species, we were able to annotate 36,927 protein-coding loci (Table 1). The genome had a Benchmarking Universal Single-Copy Orthologs (BUSCO) score of 97.7%, indicating that it is nearly complete and comparable to other high-quality grass genomes, such as *B. distachyon* (International Brachypodium Initiative 2010; current release v3.2, https://phytozome-next.jgi.doe.gov/info/Bdistachyon_v3_2) and *O. sativa* ssp. *japonica* variety KitaakeX (Jain et al. 2019; Table 1). Among the 6 grass genomes, we noted that gene density (genes per Mb) was negatively correlated with genome size, indicating that noncoding sequence accounts for much of the observed differences in genome size (Table 1; Fig. 1a).

### Synteny between grass genomes

We determined the syntenic relationships between the genomes of 6 grass species using GENESPACE (Lovell et al. 2018), which applies a multispecies orthologous gene network construction approach constrained within collinear sequence blocks. The 6 grass species include 2 perennial grasses, *B. sylvaticum* and *P. hallii*, and four annual grasses, *B. distachyon*, *B. stacei*, *S. bicolor*, and *O. sativa*. These genomes were selected because they represent both perennial and annual lifestyles, are representative of the major grass lineages, and are all very high-quality genomes that were annotated by the same JGI pipeline. *Brachypodium sylvaticum* and *B. stacei* exhibited near-perfect chromosome-scale synteny (Fig. 1b) except that *B. sylvaticum* chromosome 5 was syntenic with *B. stacei* chromosomes 5 and 10. Thus, *B. sylvaticum* chromosome 5 is similar to *B. distachyon* chromosome 4, and, as previously published, the chromosomal arrangement in *B. stacei* resulted either from the fission of a larger chromosome like the ones in *B. sylvaticum* and *B. distachyon* or by a reciprocal translocation followed by fusion of 2 ancestral chromosomes (Fig. 1). The *B. distachyon* chromosomes appear to be derived from a series of nested insertions of whole chromosomes similar to the smaller *B. sylvaticum* chromosomes into the centromeres of other chromosomes as has been previously reported (Gordon et al. 2020). Our observations also agree with cytogenetic analysis of synteny in the genus *Brachypodium* (Lusinska et al. 2019). Overall, the synteny across the 6 grasses is remarkably well conserved. This is consistent with the notion that while chromosome number may vary, gene order and telomere location have been largely conserved during the evolution of grasses from a common ancestor more than 50 MYA as reviewed in Bennetzen and Chen (2008). This highly conserved synteny can help researchers translate genes involved in domestication or other vital traits between grass species as demonstrated for barley, wheat, and sugarcane (Brueggeman et al. 2002; Griffiths et al. 2006; Le Cunff et al. 2008).

### Repetitive DNA and genome size

Sequenced grass genomes vary in size by about 6-fold. To investigate how *B. sylvaticum* fits in this diversity, we compared the composition of 6 high-quality grass genomes that vary in size by up to 3-fold (Table 1). At 358 Mb, the assembled size of the *B. sylvaticum* genome is compact but substantially larger than its close relatives *B. distachyon* (271 Mb) and *B. stacei* (234 Mb; Table 1).

To identify sources of the variation observed in genome size, we compared the proportion of DNA with different annotations (e.g. coding, intron, nongenic, and transposable element) for the 6 grass genomes (Fig. 1a; Supplementary Table 2). The amount of coding, intron, and intergenic DNA was similar in all genomes (coding sequence ranged from 31 to 42 Mb, intron sequence ranged from 43 to 57 Mb, and intergenic sequence ranged from 148 to 617 Mb). In contrast, the total amount of repetitive DNA differed dramatically with a low of 41 Mb in *B. stacei* and a high of 463 Mb in *S. bicolor* (Fig. 1a and Table 1). Repetitive DNA accounts for 89% of the 3-fold genome size difference between *B. stacei* and sorghum and 99% of the 1.5-fold size difference between *B. stacei* and *B. sylvaticum*. The largest contributor to the observed differences in repetitive DNA is LTR retrotransposons (332 Mb in sorghum vs 107 Mb in *B. stacei*), and the second largest contributor is DNA TEs. This is consistent with numerous previous studies that showed TE content was largely responsible for genome size differences (Orozco-Arias et al. 2019).

### Synteny-based orthology

We used the output of the GENESPACE pipeline to define groups of orthologous genes among the 6 grass genomes. This approach allows for construction of outgroup-constrained orthologous groups within collinear sequence alignments within both duplicated and single-copy regions. The number and the size of *B. sylvaticum* orthologous groups are similar to the other 5 grasses (Fig. 6a; Supplementary Fig. 1 and Table 3). A total of 15,075 (46.5%) *B. sylvaticum* orthologous groups were shared among all 6 species (core genes) and 20,916 (64.6%) with at least 1 other species. For the genes shared by all 6 species, 12,624 (83.7%) of them are single-copy (1:1:1:1:1:1) orthologs, 152 (1%) of them are single copy in *B. sylvaticum* but ≥ 2 copies in at least 1 other species, and the remaining 308 (2%) have at least 2 copies in *B. sylvaticum* (Supplementary File 1). One thousand two hundred and ninety-nine (5.1%) orthologous groups have members only in the *Brachypodium* species, and 9,893 (30.5%) orthologous groups were unique to *B. sylvaticum* (Fig. 6a; Supplementary Fig. 1 and Table 3).

### Transcriptomic responses to freezing and recovery

**Fig. 2.**
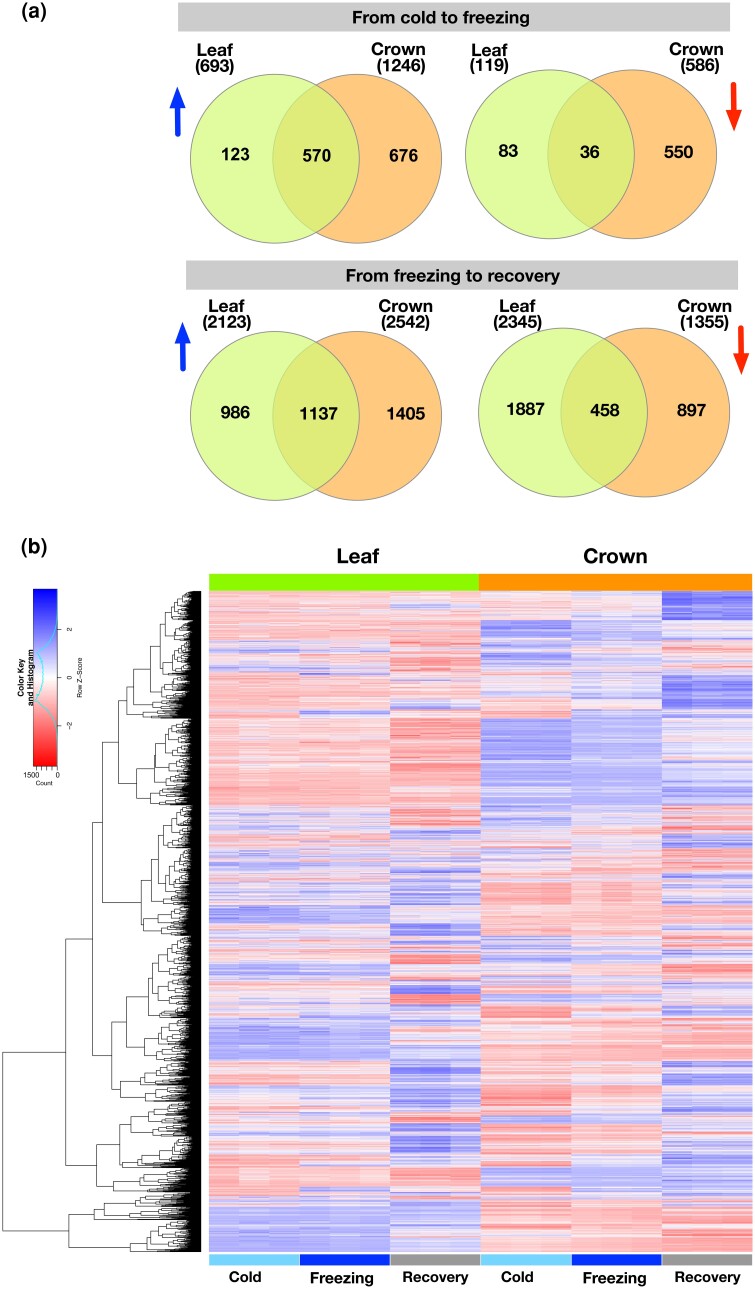
Transcriptomic responses to cold stress. a) Venn diagram of up- and downregulated genes during cold to freezing and from freezing to recovery in crowns and leaves. The blue arrows indiate upregulated genes and the red arrows indicate downregulated genes. b) Heatmap of 9,475 significantly DEGs in at least 1 treatment.

Crowns play a critical role in perennial grasses because, in addition to serving as the connection between the aerial and below-ground portions of the plant, they survive over the winter and resume the growth of new stems in the spring via the activation of axillary buds. Since many perennial grasses must survive harsh winters, crown freezing tolerance is an important trait. To better understand freezing responses and recovery in different organs of a perennial grass, we subjected *B. sylvaticum* to a freezing–recovery episode and measured gene expression at key treatment points. Plants were first treated with cold (4°C) for 14 days to allow them to acclimate. They were then subjected to freezing (−6°C) for 8 h. Next, they were moved back to 4°C for 24 h to recover and finally moved back into a growth chamber under standard warm conditions. Samples from 2 tissues (leaf and crown; see *Materials and methods* for definition) were collected at 3 stages: cold, freezing, and recovery (Supplementary Fig. 2a) and subjected to transcriptome analysis. The timepoints were selected to identify early changes in gene expression in response to freezing and recovery; however, they do not simulate an entire natural winter cycle. DEGs were identified as genes that exhibited at least a 2-fold change in transcript abundance (Wald test, adjusted *P* < 0.05) using the R package DESeq2 (Love et al. 2014). A total of 9,475 DEGs were identified that were significant in at least 1 of the comparisons: freezing vs cold and recovery vs freezing in leaf and/or crown (Fig. 2; Supplementary File 2). The freezing vs cold comparison identified 812 (up: 693; down: 119) and 1,832 (up: 1,246; down: 586) DEGs in leaf and crown, respectively, indicating that more genes were upregulated than downregulated (Fig. 2a) and that more genes responded to freezing in crowns than in leaves. The recovery vs freezing comparison identified 4,783 (up: 2,123; down: 2,345) and 3,897 (up: 2,542; down: 1,355) DEGs in leaf and crown, respectively, suggesting that more genes in leaves responded during the recovery process (Fig. 2a). Even though the plants survived the freezing challenge, a large number of genes remained differentially expressed even after 1 week under warm conditions when compared to plants prior to cold treatment (Fig. 2a). Given the obvious damage caused by freezing, the leaves appeared water soaked and flaccid after freezing, the long-lasting changes in transcription are not surprising. Previous research on cold acclimation showed that crowns can adapt to freezing and recovery more rapidly than leaves and roots in oats and wheat (Tanino and McKersie 1985; Livingston et al. 2016). Thus, the larger number of DEGs in crowns compared to leaves is consistent with previous observations.

We created a heatmap of normalized expression for the genes with DE in at least 1 comparison within each tissue (Fig. 2b). Crowns had a distinct expression pattern in response to freezing whereas leaves had similar expression profiles in both cold and freezing conditions. This is consistent with crowns responding to freezing to increase freezing tolerance.

**Fig. 3.**
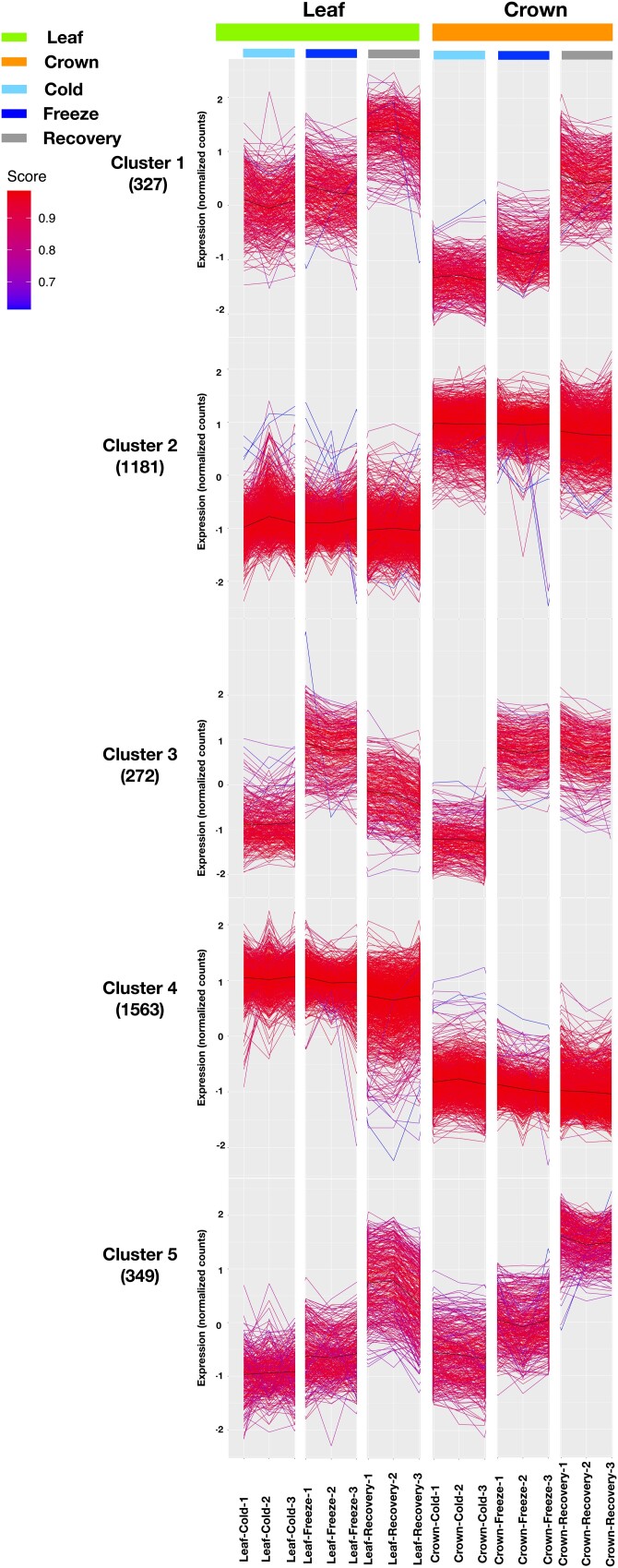
Expression analysis using *k*-mean clustering approaches with normalized counts for 3,296 DEGs with variance >1.5 and mean expression >4 in the leaf and crown under cold, freezing, and recovery conditions.

To identify coregulated genes that may be involved in freezing tolerance, we performed cluster analysis using the normalized counts from 3,296 DEGs (∼40% of DEGs) with high variance and high average expression (see *Materials and methods*). We identified 5 clusters (Fig. 3) using several approaches. Cluster 3 was particularly interesting because it contained genes that were highly induced by freezing and that induction persisted more in crowns than leaves during recovery (Fig. 3). This suggests that this cluster may contain genes that promote freezing tolerance in crowns. Examination of the gene families contained in cluster 3 revealed massive enrichment for 4 families well known to be involved in cold tolerance: WRKY (22-fold enrichment, chi square test, *P* ≤ 0.01), AP2 domain containing proteins (24-fold enrichment, chi square test, *P* ≤ 0.01), C-repeat/dehydration-responsive element binding factors (CBFs; 4-fold enrichment, chi square test, *P* ≤ 0.01), and ethylene-responsive transcription factors (ERF; 7-fold enrichment, chi square test, *P* ≤ 0.01; Supplementary Fig. 3 and Table 4). Numerous studies have demonstrated the involvement of these gene families in cold tolerance including a study in which overexpression of CsWRKY46 from cucumber in *Arabidopsis* conferred increased cold tolerance (Zhang et al. 2016). Another study showed that overexpression of a soybean WRKY, GmWRKY21, in *Arabidopsis* enhanced freezing tolerance (Zhou et al. 2008). In *Verbena bonariensis*, VbWRKY32 was shown to upregulate cold/freezing response genes, and overexpression of this gene improved freezing tolerance (Wang et al. 2019). The *Brassica campestris* gene *BcWRKY46* is strongly induced by low temperature and ABA and has been shown to induce genes in the ABA signaling pathway to improve cold tolerance (Wang et al. 2012a). AP2, ERF, and CBFs all belong to the AP2/ethylene-responsive element binding factor (EREB) domain family. They regulate genes involved in diverse biological processes such as growth, development, hormone, and stress responses (Dietz et al. 2010; Gibbs et al. 2011; Licausi et al. 2011; Chandler 2018). Among these, DREB1s (DREB-A1 subgroup) contain several CBFs that play major roles in freezing tolerance (Chinnusamy et al. 2003).

### Expression dynamics under heat, salt, and water deprivation stresses

**Fig. 4.**
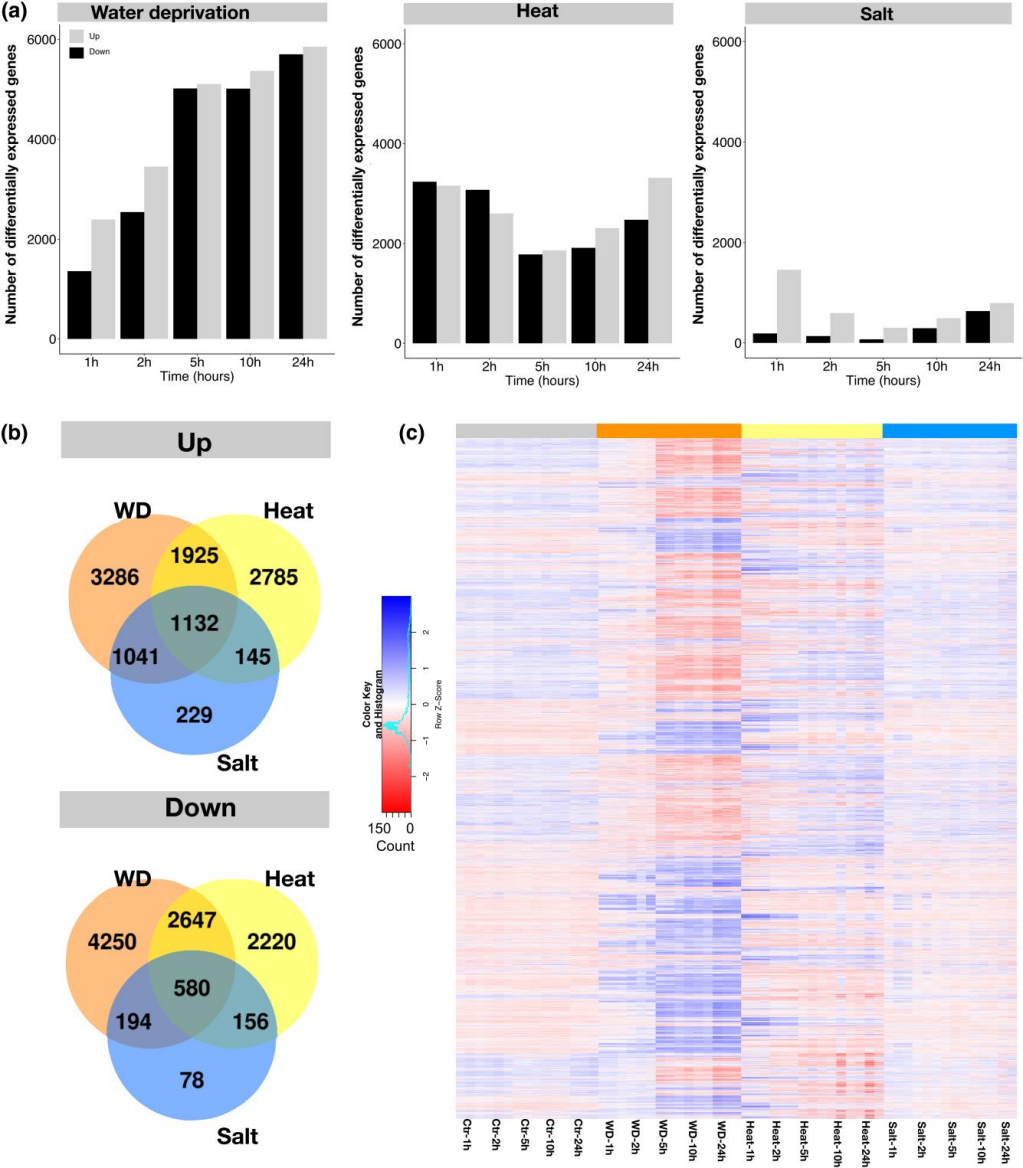
Transcriptomic response to stress. a) The number of up- and downregulated genes at different timepoints under water deprivation, heat, and salt stresses. b) Venn diagram of up- and downregulated genes under water deprivation, heat, and salt stresses (combined timepoints for each treatment). c) Heatmap of 17,184 genes that were significantly differentially expressed genes in at least 1 timepoint and treatment.

Abiotic stresses, such as heat, freezing, drought, and salt limit plant growth and yield in many locations. Understanding the molecular mechanisms by which plants respond to and tolerate abiotic stress can help lead to rational approaches to engineer or breed improved crops. This may be particularly important for the perennial grasses emerging as biomass crops (e.g. switchgrass and *Miscanthus*) for 3 reasons. First, they are largely undomesticated, and there is tremendous potential for improving their stress tolerance. Second, to decrease competition with food crops, these emerging crops will be grown on marginal land that is subjected to more abiotic stress, especially drought, heat, and salt, than crops grown on prime agricultural land. Third, in order to maximize net energy production, biomass crops must be grown with minimal agricultural inputs (e.g. irrigation and fertilization). Since it is difficult to conduct controlled experiments with biomass grasses, understanding the molecular response in *B. sylvaticum* can provide information that can be used to develop approaches to breed and/or engineer more stress-tolerant biomass crops. We measured transcriptomic responses to 3 abiotic stresses (water deprivation, heat, and salt) at 5 timepoints (1, 2, 5, 10, and 24 h) selected to capture early responses to the stresses (Fig. 4). We use the term water deprivation rather than drought because our assay to impose water stress, removing plants from the soil, was designed to examine the initial stages of lack of water rather than the longer-term effects associated with more traditional drought treatments. While the way in which we imposed these stresses is not the same as long-term stress in the field, we used these methods because they are highly reproducible and allow us to investigate early responses. In addition, these methods were employed by the JGI Gene Atlas project which will allow our data to be compared to several species (Sreedasyam et al. 2023).

Principal component analysis and *k*-mean clustering of gene expression showed that samples from each stress treatment largely clustered together except for one 24-h salt-treated sample which was a clear outlier by both measures and was removed from further analysis. The heat and water deprivation samples formed distinct groups on the principal component analysis (PCA) plot and *k*-mean clustering analysis indicating that the overall responses were dramatic and distinct. In contrast, the salt-treated plants clustered close to control samples in both the PCA and *k*-mean clustering, indicating a much weaker response. This may indicate that the concentration of the salt solution was not enough to cause severe stress. Indeed, we did not observe an increase in the expression of known salt tolerance–related genes (e.g. BsHKT8 and BsNHX1) that have previously been shown to be upregulated in *B. sylvaticum* in response to salt stress (Sade et al. 2018). Overall, 10,543 genes (28.55% of all genes) were significantly upregulated and 10,125 (27.41% of the total annotated genes) were significantly downregulated in response to at least 1 abiotic stress (Fig. 4a and b). In response to heat, water deprivation, and salt stresses, 5,987, 7,384, and 2,547 were significantly upregulated and 5,603, 7,671, and 1,008 were significantly downregulated, respectively (Fig. 4b).

While the expression of many genes was altered in response to all 3 stresses, they differed in their temporal dynamics. The response to heat treatment was biphasic with many genes responding during the first 2 timepoints (1 and 2 h) followed by a rapid drop in the number of DEGs at 5 h and then a gradual increase in the number of DEGs during the later time points (10 and 24 h; Fig. 4a). This is consistent with previous research in numerous systems including *Drosophila melanogaster* (Sorensen et al. 2005), *A. thaliana* (Swindell et al. 2007), barley (Mangelsen et al. 2011), and *Caenorhabditis elegans* (Jovic et al. 2017). Unlike the response to heat stress, the response to both water deprivation and salt exhibited a steady increase in the number of responding genes throughout the time course. This agrees with previous observations of drought stress in maize (He et al. 2020) and sorghum (Vohra et al. 2021) as well as in *Populus* (Liu et al. 2019) and *Cenostigma pyramidale* in response to salt stress (Frosi et al. 2021).

**Fig. 5.**
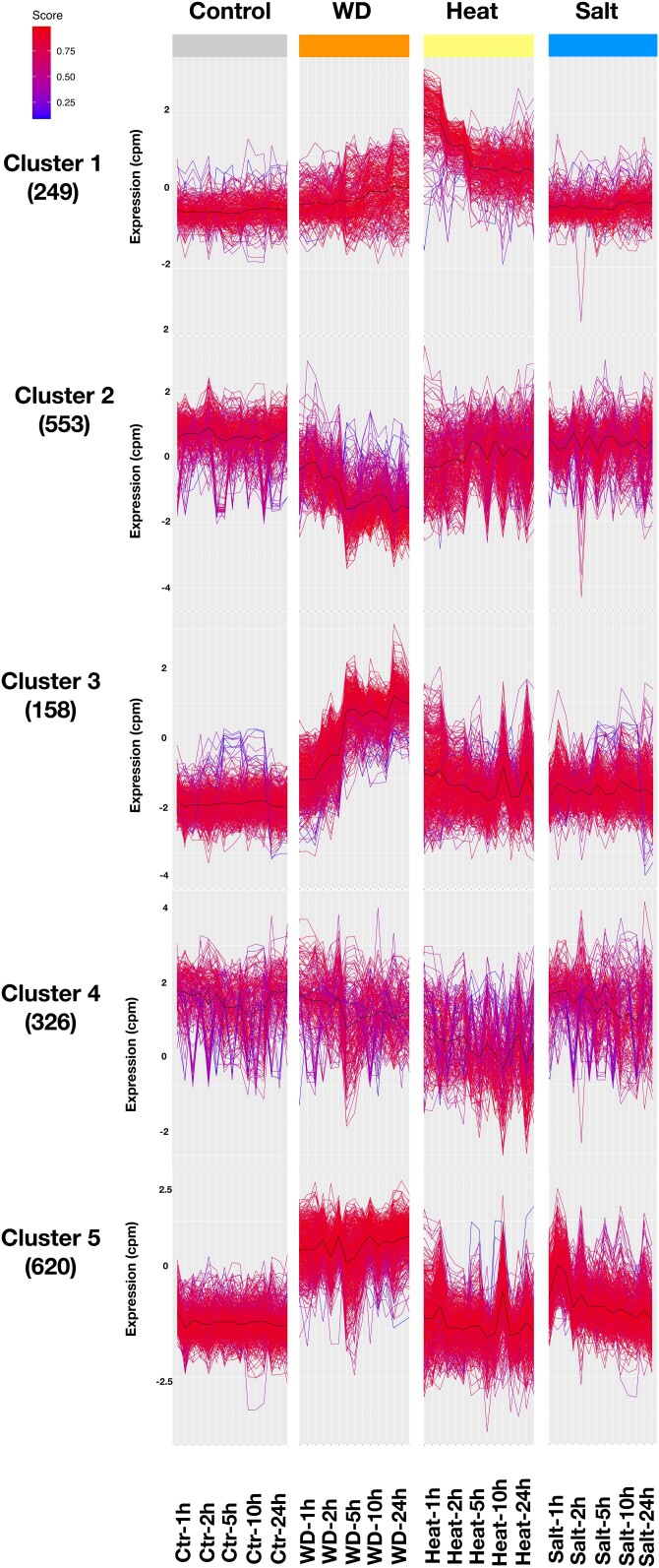
Expression analysis using *k*-mean clustering approaches with normalized counts for the 1,911 DEGs with variance >1.5 and mean expression >4 in under heat, water deprivation (WD), and salt stress conditions.

To see if specific gene clusters respond differently to the stresses, we examined 17,184 genes with significant DE during at least 1 timepoint for at least 1 treatment. For each of the genes, we calculated the average expression abundance (normalized by counts per million (cpm); Supplementary File 3). Then we picked genes with high variance (>1.5) and high mean (>4 cpm) expression abundance for further analysis. In total, 1,911 genes passed those criteria. We performed *k*-mean clustering to place the genes into 5 clusters, each with a different expression pattern (Fig. 5). Five clusters were chosen based on prior optimization. Cluster 1 and cluster 5 show patterns that are typical of heat and drought responses, respectively. Genes in cluster 1 show a rapid increase in expression 1–2 h after heat treatment followed by a decrease in expression before leveling off at later timepoints (Fig. 5; Supplementary Fig. 4). This pattern is consistent with the behavior of heat shock proteins (HSPs) and other rapid heat response genes that have been observed in many organisms (Schulze et al. 2005). Enrichment analysis showed cluster 1 is enriched in HSPs (150-fold enrichment, chi square test, *P* ≤ 1 × 10^−5^). Note that the enrichment analysis included all the *B. sylvaticum* HSPs and other chaperones (as defined by Chen and Li 2017) that were induced in *B. distachyon* by heat stress (Supplementary Table 6). HSPs are molecular chaperones that help other proteins maintain their native conformation, thus improving protein stability under stress (Wahid et al. 2007). Extensive evidence indicates that HSPs play important roles in thermotolerance and that some specific HSPs are causally involved in the capacity to acquire thermotolerance, for example HSP101 in maize (*Zea mays* L.) and *Arabidopsis* (Hong and Vierling 2001; Nieto-Sotelo et al. 2002), HSP90 in *Arabidopsis* (Ludwig-Müller et al. 2020), HSP70 in tobacco (*Nicotiana tabacum* L.; Cho and Choi 2009), and Small heat shock proteins in maize and creeping bentgrass (Heckathorn et al. 1998; Luthe et al. 2000).

Genes in cluster 5 showed much higher expression under water deprivation at all timepoints (Fig. 5; Supplementary Fig. 4). The annotations of the cluster 5 genes were enriched for genes belonging to WRKY and AP2 gene families (10-fold enrichment, *P* < 1 × 10^−5^, chi square test and 7-fold enrichment, *P* < 1 × 10^−5^, chi square test, respectively; Fig. 5; Supplementary Table 5). The enrichment observed in WRKY and AP2 domain gene families is consistent with previous reports that these gene families play an important role in drought response in several plants (Bakshi and Oelmuller 2014). For example, the drought-responsive WRKY transcription factor genes *TaWRKY1* and *TaWRKY33* from wheat confer drought tolerance (He et al. 2016). The *Medicago truncatula* gene *MtWRKY76* responds rapidly to drought stress, and its overexpression increases drought tolerance (Liu et al. 2016). In *Arabidopsis*, *WRKY46* has been shown to regulate responses to drought stress (Ding et al. 2014).

### PS of protein-coding genes

**Fig. 6.**
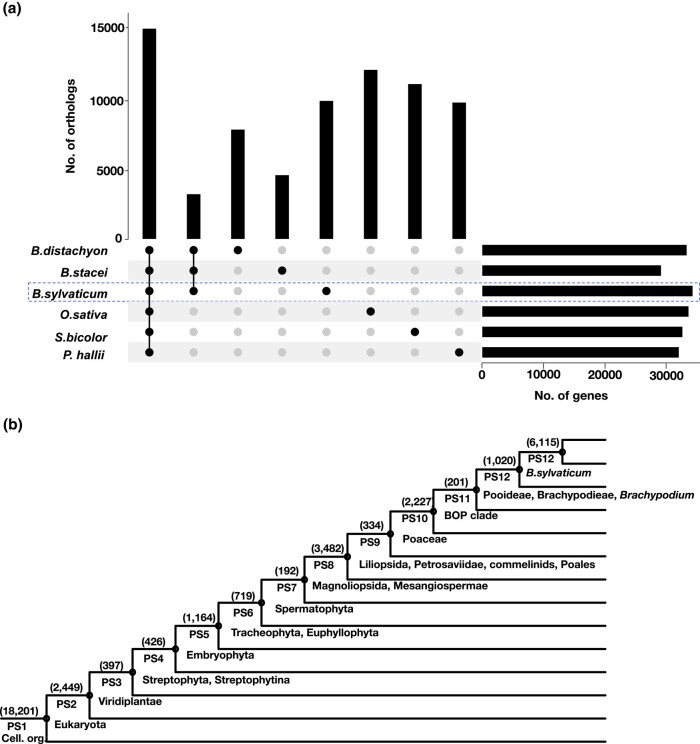
Comparative genomics and phylostratigraphic analysis. a) Partial upset plot showing the number of orthologs shared by all 6 grasses, *Brachypodium* species, and species-specific orthologs. The full upset plot can be found in Supplementary Fig. 1. b) Phylostratigraphic map of *B. sylvaticum*. Numbers in parentheses denote the number of genes per phylostratum (PS1–PS13). Cell. org., cellular organisms described by PS1.

The timing of the origin of any gene can be deduced by the length of time that has passed since a group of species containing versions of that gene last shared a common ancestor. This timing has been termed the phylostratum of that gene in a nod to the use of the geological strata to date fossils (Domazet-Lošo and Tautz 2010). To place the DEGs from clusters 1 and 5 into a broader evolutionary context, we determined the PS of all *B. sylvaticum* genes. Using the method developed by Drost et al. (2015), the 36,927 protein-coding genes of *B. sylvaticum* were assigned to 13 PS going back 3.6 billion years (Fig. 6; Supplementary File 4) as defined in Wang et al. (2018b). These PS range in age from the oldest group, PS1, that contains genes dating back to the last common ancestor between prokaryotes and eukaryotes (ca. 3,556 MY) to the youngest group, PS13, that contains genes found only in *B. sylvaticum* (10 MY). Over half of the *B. sylvaticum* genes (57%) originated from the 3 most ancient PS (cellular organisms, Eukaryota, and Viridiplantae), which is similar to what was observed in rice (46.3%; Wang et al. 2018b) and *Arabidopsis* (64.9%; Quint et al. 2012; Lei et al. 2017). GO term analysis showed an enrichment for genes with catalytic activity (GO:0003824). Presumably, this is due to ancient conservation among the genes that control basic metabolic processes. The proportion of genes unique to 1 species (16.6%) is much higher than in *O. sativa* (0.34%; Wang et al. 2018b) and *A. thaliana* (6.93%; Quint et al. 2012; Lei et al. 2017); however, this may simply reflect differences in annotation methods.

Interestingly, the genes in cluster 5 were enriched in genes belonging to PS4 which makes sense because cluster 5 genes are responsive to water stress and PS4 is coincident with the emergence of land plants when plants first encountered frequent water stress. A similar enrichment for PS4 genes was observed for the genes in clusters 3 and 4, which also showed altered expression during water deprivation. In addition, clusters 3 and 4 also showed enrichment for genes in PS6 (Tracheophyta) and PS7 (Spermatophyta). Taken together, these results suggest that many genes responsive to water deprivation originated from early evolutionary stages when plants evolved the ability to colonize land.

Our results continue the development of resources that facilitate the use of *B. sylvaticum* as a model perennial grass. The high-quality genome produced in this study will undoubtedly be widely used by researchers conducting research with *B. sylvaticum.* Our transcriptomic analyses identified candidate genes involved in stress responses and will hopefully serve as a seed for more a comprehensive *B. sylvaticum* gene atlas. We demonstrated that crowns are more responsive to cold than leaves. This may be due to the perennial nature of *B. sylvaticum* which highlights the need for a model perennial grass.

## Data Availability

All data used in this publication are freely available and can be accessed through the supplemental files or the links therein. Specifically, the raw PacBio sequence data for reference genome assembly can be downloaded from NCBI, BioProjectID PRJNA786589. The Illumina sequences from F_2_ parental lines can be downloaded from NCBI, BioProjectID PRJNA786589. The links to download raw RNA-seq data and the BioProject IDs for gene annotation and stress experiments are available in Supplementary Table 1. The assembled genome and gene annotation can be downloaded from Phytozome 13 (https://phytozome-next.jgi.doe.gov/info/Bsylvaticum_v1_1). All of the scripts for data analysis of this manuscript can be seen in https://github.com/lilei1/B_sylvaticum. Supplemental material available at figshare: https://doi.org/10.25387/g3.23519004.
